# Dosimetric and mechanical equivalency of Varian TrueBeam linear accelerators

**DOI:** 10.1002/acm2.13058

**Published:** 2020-10-18

**Authors:** Mohammed Ghazal, Lars Södergren, Mathias Westermark, Julia Söderström, Tobias Pommer

**Affiliations:** ^1^ Department of Medical Radiation Physics and Nuclear Medicine Karolinska University Hospital Stockholm Sweden; ^2^ Department of Hematology, Oncology and Radiation Physics Skåne University Hospital Lund Sweden

**Keywords:** beam‐matching, DLG and jaw calibration, energy‐matching

## Abstract

**Purpose:**

To investigate and improve the level of equivalency of Varian TrueBeam linear accelerators (linacs) in energy‐, dosimetric leaf gap‐ (DLG) and jaw calibration.

**Methods:**

Eight linacs with four photon energies: 6 MV, 6 MV FFF, 10 MV FFF, and 15 MV, and three electron energies (on two linacs): 6, 9, and 12 MeV were commisioned and beam‐matched. Initially, symmetry of lateral profiles was calibrated for maximum field size. Energy‐matching was then performed for photons by adjusting diagonal profiles at maximum field size and depth of maximum dose to coincide with the reference linac, and for electrons by matching the range at percentage depth of ionization of 90%, 80%, and 50%. Calibration of DLG was performed for 6 MV and evaluated among the linacs. The relationship between DLG and the Gap value was investigated. A method using electronic portal imaging device (EPID) was developed and implemented for jaw calibration.

**Results:**

Symmetry calibration for photons (electrons) was within 1% (0.7%), further improving the vendor's acceptance criteria. Photon and electron energy‐matching was within 0.5% and 0.1 mm, respectively. Calibration of DLG was within 0.032 mm among the linacs and utilizing the relationship between DLG and the Gap value resulted in an empirical calibration method which was implemented to simplify DLG adjustment. Using EPID‐based method of calibration, evaluation of the jaw‐positioning among the linacs for 30 cm × 30 cm field size was within 0.4 mm and in the junction area within 0.2 mm. Dose delivery error of VMAT‐plans were at least 99.2% gamma pass rate (1%, 1 mm).

**Conclusions:**

High level of equivalency, beyond clinically accepted criteria, of TrueBeam linacs could be achieved which reduced dose delivery systematic errors and increased confidence in interchanging patients among linacs.

## 1 INTRODUCTION

A high demand for radiotherapy treatments requires an effective workflow, especially in clinics with several linear accelerators (linacs). A key component in achieving high efficiency is the ability to move patients to another linac without the need to adjust the treatment plan. This is accomplished by having dosimetrically and mechanically equivalent linacs, that is, linacs that are beam‐matched.^1^, ^2^, ^3^, ^4^, ^5^, ^6^, ^7^ When commissioning new linacs, they are often beam‐matched by the vendor upon delivery. However, it has been shown that vendor specification might not be strict enough to ensure optimal matching.^1^, ^3^ According to vendor specifications, beam‐matching refers to energy‐matching. However, as the level of complexity in treatments increases, the importance of other parameters increase. These include dosimetric leaf gap (DLG) which determines the positioning of the multileaf collimators (MLC) and is highly relevant for all dynamic treatments, and jaw positioning.^8^, ^9^, ^10^ Ultimately, achieving nominally matched linacs enables moving patients among the linacs in case of malfunction, service etc. and hence enhancing the flexibility and the efficiency of the workflow in the clinic. Moreover once it is established that the linacs are nominally matched, only one set of beam‐data is needed for modelling the beam in the treatment planning system (TPS).^11^

Several studies have presented beam matching techniques and corresponding results for linacs of different vendors, models, and energies.^2^, ^3^, ^7^, ^12^, ^13^, ^14^ Also, multi‐institutional studies have been conducted to compare beam matching prestanda.^1^, ^11^, ^15^ However, there is a lack of a complete set of data for energy matching, DLG‐, and jaw position calibration for a significant number of linacs within one clinic which can serve as a reference for other clinics in the process of beam‐matching TrueBeam linacs.

Eight linacs were installed at the radiotherapy department at Karolinska University Hospital (Stockholm, Sweden) with four photon energies: 6 MV, 15 MV, 6 MV FFF, and 10 MV FFF, and, on two linacs, three electron energies: 6, 9, and 12 MeV. The linacs were installed two at a time over a six months period. The linacs' components were all of the same series and they were factory‐matched upon delivery. A group of five medical physicists (the authors of this article) were tasked with commisioning the linacs clinically within seven months of the installation of the first linac. The purpose of this work was to investigate the highest level of agreement among TrueBeam linacs, and to present the methodologies to achieve it. Higher equivalency among beam‐matched linacs reduces dose delivery systematic errors as well as increases the confidence in swapping patients among the linacs, having one set of beam‐data in the TPS. To the best of our knowledge, this is the first investigation of beam‐matching for more than three TrueBeam linacs at the same institution, as well as including DLG calibration and jaw calibration in the beam matching process. The high number of linacs in this work, coupled with measurements being conducted during a short time frame using the same equipment and methodologies, qualify the results as a reliable and credible reference for other clinics undergoing a similar task.

## 2 MATERIALS AND METHODS

### 2.A Symmetry calibration and energy‐matching

Symmetry calibration for photons (electrons) was performed in water using the IBA Blue Phantom 2 and IBA Compact Chamber CC13 (Table 1), for all energies by measuring 40 cm × 40 cm (25 cm × 25 cm) lateral profiles in in‐ and crossline directions. The measurements setup for photons was performed at source‐to‐surface distance (SSD) = 90 cm and depth in water = 10 cm. For electrons, the setup was SSD = 100 cm and depth in water equals that of maximum depth dose d_max_. The aim was to adjust the beam steering in order to obtain the best symmetry value possible, regardless of whether profiles were already within the acceptance criteria from the factory.

**Table 1:** Information about IBA detectors which were used in this work.

Name	Type of detector	Effective volume [cm^2^]	Inner diameter [mm]	Type of measurements
FC65‐G	Farmer ionization chamber	0.65	6.2	DLG and photon reference dosimetry
PPC40	Plane Parallel ionization chamber	0.40	16	Electron depth dose and reference dosimetry
CC13	Compact ionization chamber	0.13	6	Photon and electron lateral profiles, Photon depth dose

A linac's energy quality is commonly characterized by the Tissue Phantom Ratio (TPR_20,10_).^16^ Consequently, TPR_20,10_ measurements can be used for energy‐matching among linacs. However, a more comprehensive method of photon energy‐matching is measuring and matching diagonal dose profiles at d_max_, after symmetrizing the lateral dose profiles. This method offers more information about the beam profile of a specific energy compared to TPR_20,10_.^17^

After obtaining optimized symmetry values the energy‐matching of each photon energy was performed. The first installed linac was considered the reference to which all other linacs were matched. A pair of diagonal dose profiles were measured with SSD = 90 cm and depth in water = depth of maximum dose (i.e., 1.5 cm for 6 MV, 2.5 cm for 10 MV, 3.0 cm for 15 MV). The aim was to minimize the difference between the reference and the actual linac in the region above 80% and 60% of the central axis dose for flattened and unflattened beams, respectively (the latter roughly corresponding to 80 % of the full width half maximum (FWHM) value). The electron energies were matched by measuring the percentage depth of ionization (PDI) at SSD = 100 cm for 10 cm × 10 cm field size and adjusting the energy so that the electron range at the percentage depth of ionization 90%, 80%, and 50% (R90, R80 and R50) were tuned with the reference linac. Priority was given to match the electron range at the central axis of the PDI rather than the lateral dose profile for the largest field size.

### 2.B Reference dosimetry

Reference dosimetry was performed in accordance with TRS‐398 Code of Practice (CoP)^16^ for flattened photon beams and electron beams, and TRS‐483^18^ CoP for unflattened photon beams. TPR_20,10_ was measured on all linacs and an average value was used to readout the appropriate k_Q,Q0_. Note that for unflattened beams, determining TPR_20,10_ required a correction to get an equivalent uniform field corresponding the reference field of 10 cm × 10 cm as described in TRS‐483.^18^ Similarly for electron beams, R50 was determined for both linacs and an average was used for the readout of the k_Q,Q0_. The IBA FC65‐G Farmer‐type ionization chamber and IBA PPC40 plane parallel ionization chamber were used for photons and electrons, respectively (Table 1). An external audit of the reference dosimetry calibration was afterwards performed by Medical Physics Services Intl. Ltd., Cork, Ireland.

### 2.C Dosimetric Leaf Gap calibration

Dosimetric Leaf Gap (DLG) is a key parameter in matching linacs, most importantly for dynamic treatments.^5^, ^7^, ^19^ Dosimetric Leaf Gap calibration require submillimeter precision and having many linacs to be matched in a clinic require optimizing the DLG value so it can be achieved within a certain tolerance by all linacs. Careful selection of the DLG value, calibration methodology and level of tolerance is essential. The linacs were all equipped with Millenium 120‐leaf MLCs. The DLG was determined by placing the IBA FC65‐G (Table 1) in the central axis of the field at 10 cm depth and SSD 90 cm and measuring a set of sliding window dynamic plans with MLC gap widths ranging from 2 to 20 mm. Scoring the charge for each measurement, an extrapolation was done for zero dose and the DLG value was calculated.^20^, ^21^ The “Gap” value is the physical distance between the opposite leaves when they are completely closed, and it was modified to adjust the DLG to the desired value. The reference linac was set to 1.4 mm DLG and other linacs were calibrated as close as possible to that value. A simple empirical mathematical model was developed to help calculate the appropriate Gap value to achieve the desired DLG value. The calibration of DLG was performed using 6 MV as it is the energy most used for dynamic treatments and the DLG for the remaining energies were simply measured and verified to be consistent among the linacs.

**Fig. 1:**
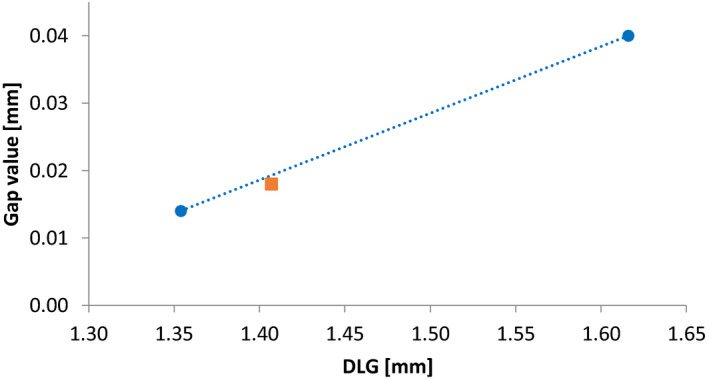
Gap values as function of measured DLG for 6 MV beam. The dotted line is a linear model of two measurements (blue circles) and the orange square is measured DLG resulting from the model‐based estimation of the Gap value for 1.4 mm DLG.

It was observed that for each linac, a linear fit of Gap value as a function of DLG could be modelled after two measurements, and the *k* and *m* parameters were used to calculate a Gap value which provide the desired DLG (Fig. 1). Using this method, it often took only three measurements to calibrate a linac to the desired DLG value.

### 2.D Jaw position calibration

The position of the jaws is initially calibrated by the vendor’s installer using the light field, after it has been verified that the radiation‐ and light fields are consistent with each other. This method has uncertainties regarding visual estimation and radiation‐ and light field agreement.^6^ For TrueBeam linacs, several publications have suggested using the Electronic Portal Imaging Device (EPID) to evaluate and calibrate the jaw position.^22^, ^23^ It has been shown that using EPID results in higher precision and reproducibility in the calibration of the jaw position which candidate the method for beam‐matched linacs.

During commissioning, it was observed that small inaccuracies in the jaw position for 20 cm × 20 cm fields could result in an over‐ or underdosage of up to 15% in the junction region, that is, the zero position of the asymmetric jaw position. In order to achieve a higher precision an EPID‐based method was developed. It utilizes the position sensor readout (PRO‐value) which is provided by the TrueBeam workstation, and the Portal Dosimetry Software (Varian Medical systems) which enables users to simulate corrections in the acquired images. The method is thoroughly explained in Appendix A.

### 2.E Beam‐match verification

To verify the beam‐matching, a set of water profile measurements with different geometries than those used during initial matching were performed on each linac and compared to those of the reference linac using purpose‐written MATLAB‐code. The verification measurement geometries for photons (electrons) consist of PDD and lateral dose profiles for 5 cm × 5 cm, 10 cm × 10 cm, 20 cm × 20 cm, and 40 cm × 40 cm (6 cm × 6 cm, 10 cm × 10 cm, 15 cm × 15 cm, 20 cm × 20 cm, and 25 cm × 25 cm). Evaluation was done using a global gamma index with 2 % and 2 mm gamma criteria. A set of output factors (OF), wedge factors and applicator factors were also measured and compared. Finally, evaluation of the dose delivery error was done by optimizing one clinical volumetric modulated arc therapy (VMAT) head & neck plan each for the 6 MV, 6 MV FFF, and 10 MV FFF energies. The plans were delivered to a dosimetric phantom (Scandidos Delta4 Phantom+) on all the linacs. For each measurement, dose and setup corrections were applied from an open field measurement. The measured dose on the reference linac was used as reference in a gamma evaluation, with 1%, 1 mm and 2%, 2 mm gamma criteria.

## 3 RESULTS

### 3.A Symmetry calibration and energy‐matching

**Fig. 2:**
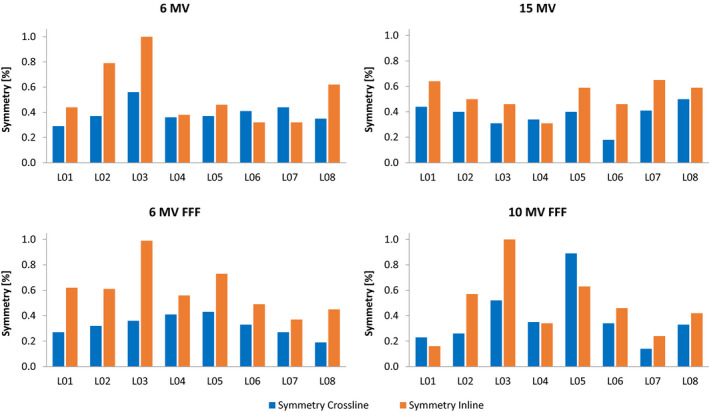
Symmetry after calibration of lateral dose profiles for 40 cm × 40 cm fields measured on eight linacs (L01‐L08).

Figures 2 shows the symmetry values after calibration of each linac for all photon energies. All values are within 1 %. Electron symmetry calibration resulted in values below 0.7%.

**Fig. 3:**
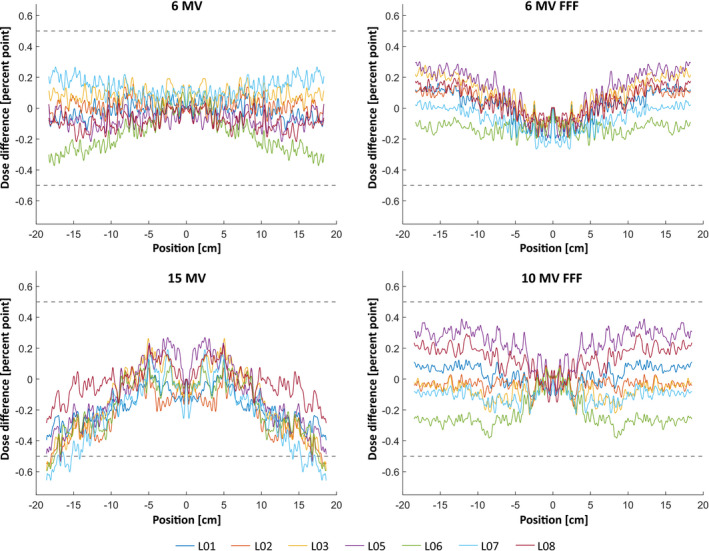
Dose difference of diagonal dose profiles for 40 cm × 40 cm fields between seven linacs and the reference linac (L01‐L08; L04 being the reference). The profiles were symmetrizied and normalized to the central axis dose for each linac before calculating the difference.

Figure 3 shows the dose difference between the reference and the seven other linacs for all photon energies for 40 cm × 40 cm field size diagonal profiles at d_max_. The displayed region of the profiles are above 80% and 60% for flattened and unflattened beams, respectively. The difference is calculated after symmetrization and normalization to the central axis dose. The maximum difference is below 0.5% except for 15 MV in the region 15 cm off the central axis. The difference in R90, R80, and R50 between the two linacs with electron energies is at most 0.1 mm.

### 3.B Reference dosimetry

**Fig. 4:**
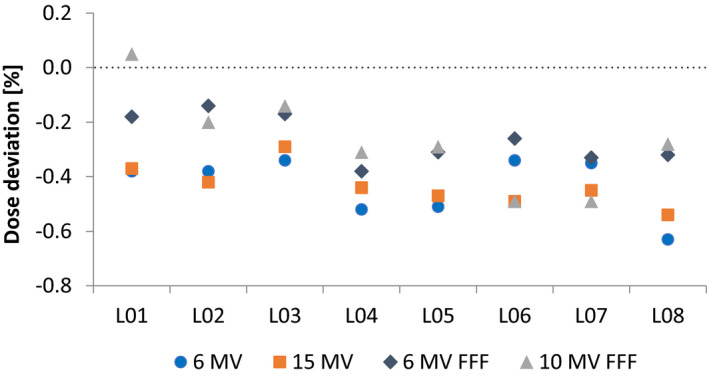
Photon dose output deviation between measurements performed by the clinic and by an external audit for eight linacs (L01–L08).

The reference dosimetry audit of photon energies is summarized in Fig. 4 where the difference between measurements performed by the institution and the external audit is presented. Except for one measurement, all audit results showed lower dose output compared with the institutional results. These deviations are attributed to calibration coefficient difference since both parties used their own equipment with different traces of calibration. Furthermore, the differences were at most 0.6% resulting in acceptable outcome of the reference dose audit. The audit of the electron energy calibration is presented in Table 2 where it is observed that no difference above 0.6% was present.

**Table 2:** Electron dose output deviation (*Dev*) between measurements performed by the clinic and by an external company for two linacs.

Linac	Dev_6 MeV_ [%]	Dev_9 MeV_ [%]	Dev_12 MeV_ [%]
TrueBeam 1	0.36	0.58	0.24
TrueBeam 2	0.21	−0.22	−0.26

### 3.C Dosimetric Leaf Gap calibration and consistency

**Fig. 5:**
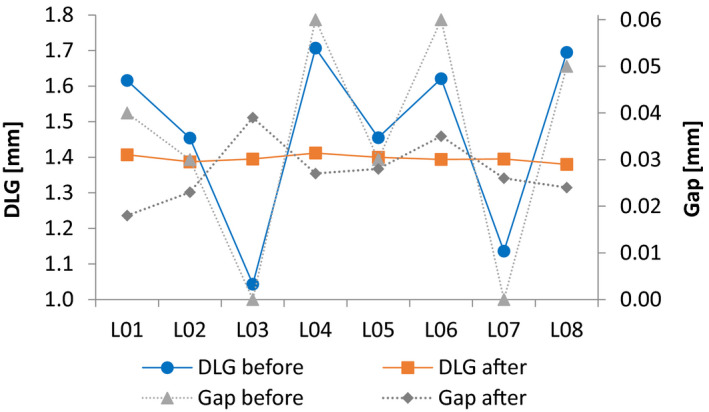
DLG values for 6 MV beams before and after calibration, with corresponding Gap values for eight linacs (L01–L08). The connecting lines are illustrative.

The DLG before and after calibration with corresponding Gap values for 6 MV are shown in Fig. 5. A clear pattern relating the Gap values and the DLG is visible before the calibration. However, this pattern diminishes after calibration, when the variations are less pronounced. Table 3 shows a comparison of DLG among the linacs after calibration where best match is for 6 MV because the calibration is performed using this energy. The other energies are measured for verification reasons.

**Table 3:** Comparison of DLG among eight linacs which were calibrated at 6 MV aiming towards a value of 1.4 mm. The comparison is in terms of minimum‐ (*Min*), maximum‐ (*Max*), median‐ and mean values.

	Energy	Min [mm]	Max [mm]	Median [mm]	Mean [mm]
DLG	6 MV	1.380	1.412	1.404	1.405
	15 MV	1.527	1.599	1.566	1.560
	6 MV FFF	1.221	1.282	1.248	1.250
	10 MV FFF	1.429	1.497	1.438	1.449

### 3.D Jaw position calibration and consistency

**Fig. 6:**
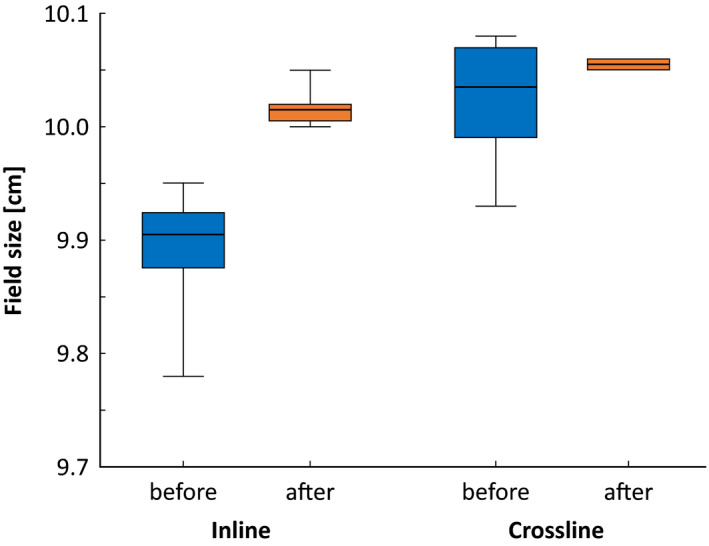
Field sizes of 10 cm × 10 cm 6 MV fields measured on eight linacs in water, before and after calibration. The boxes represent 25%–75% quartiles, the black lines in the boxes represent the median and the bars represent the range.

Figure 6 shows field sizes for 10 cm × 10 cm measured in water in 6 MV beams among the eight linacs, before and after the inhouse EPID‐based calibration. The jaws were initially calibrated by the vendor's using the light field. It is clear that this results in a large disparity in field sizes among the linacs and improved results are acquired after institutional calibration. Table 4 shows the residual over‐ or underlap at the field junction and the field size after the calibration of jaws with maximum difference of 0.2 and 0.4 mm, respectively.

**Table 4:** Comparison among eight linacs of 30 cm × 30 cm field sizes and jaw‐position accuracy at zero‐position, after calibration using EPID for 6 MV.

	Jaw	Min	Max	Median	Mean
Field size [mm]	X	299.7	300.1	299.9	299.9
	Y	299.9	300.3	300.0	300.1
Field junction [mm]	X1X2	0	0.2	0.1	0.1
	Y1Y2	0	0.2	0.2	0.2

### 3.E Beam‐match verification

**Fig. 7:**
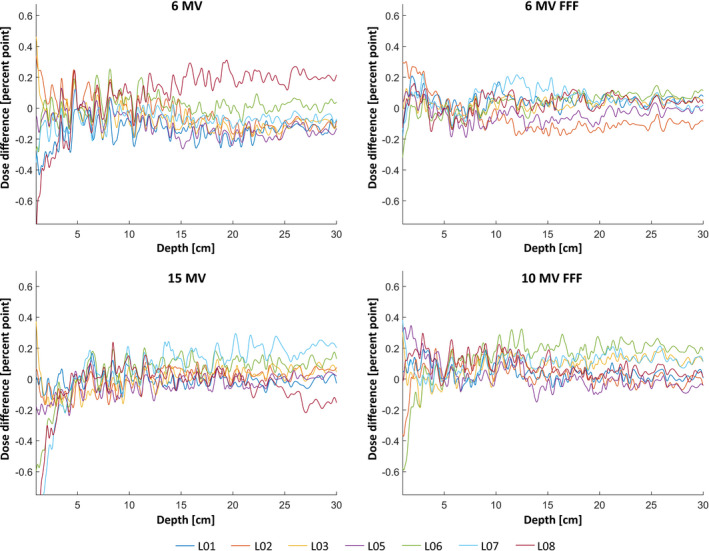
Dose difference of PDD for 10 cm × 10 cm fields between seven linacs and the reference linac (L01–L08; L04 being the reference). Note that the depth is shown in the range of 1 to 30 cm.

An overview of the comparison among the linacs in TPR_20,10_ is presented in Table 5 where the largest difference is 0.5%. Percentage depth dose difference among the seven linacs against the reference is presented in Fig. 7 where the largest difference is below 0.3 % excluding the build‐up region.

**Table 5:** Comparison among eight linacs in tissue phantom ratio (TPR_20,10_) in terms of maximum‐ (*Max*), minimum‐ (*Min*), median‐ and mean values.

	Energy	Min	Max	Median	Mean
TPR_20,10_	6 MV	0.664	0.666	0.665	0.665
	15 MV	0.760	0.762	0.761	0.761
	6 MV FFF	0.631	0.634	0.633	0.633
	10 MV FFF	0.706	0.709	0.707	0.707

**Fig. 8:**
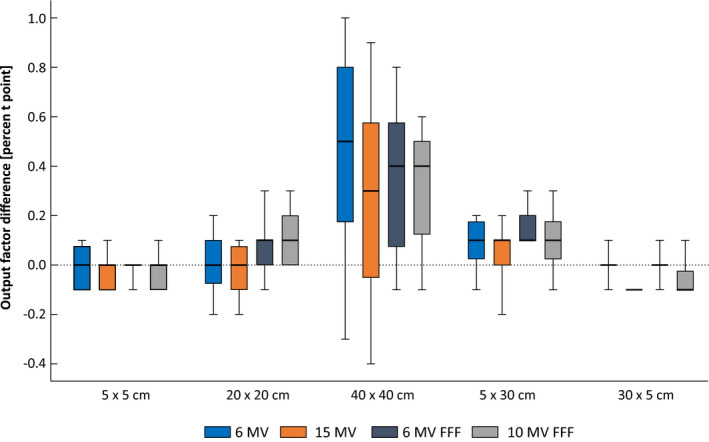
Difference in output factor between seven linacs and the reference linac for different geometries.

**Fig. 9:**
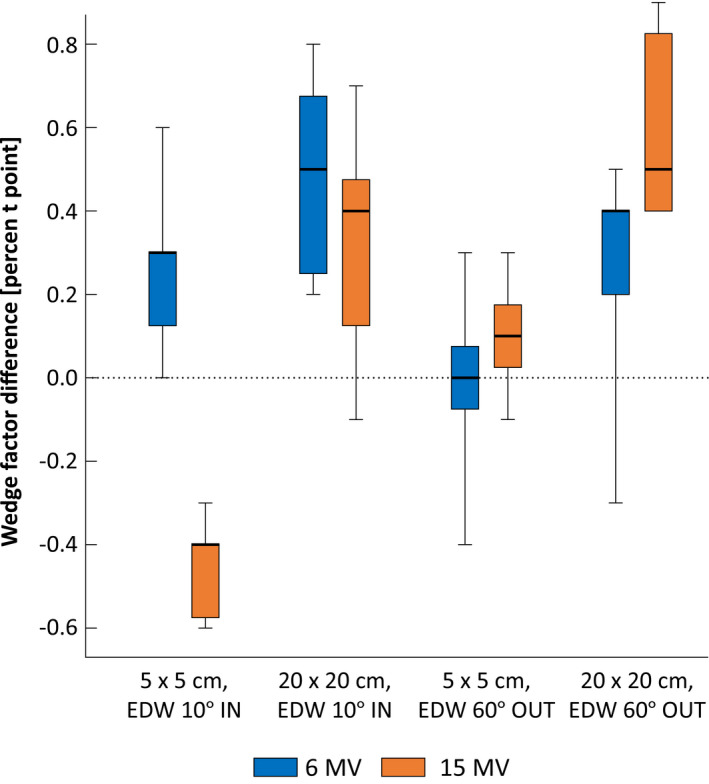
Difference in wedge factor between seven linacs and the reference linac for different geometries.

The water profile verification measurements contain a large amount of data, which is why only the mean value of the global gamma evaluation index (2% and 2 mm) for all linacs is reprted. Evaluating the verification measureents, excellent agreement is observed with the mean gamma index being 99.6% for photons and 98.2% for electrons. The differences in OF between seven linacs against the reference linac where all within 1% with OF for 40 cm × 40 cm showing the largest difference (Fig. 8). Differences in wedge factors measured in combinations of 5 cm × 5 cm and 20 cm × 20 cm with 10° and 60° are presented in Fig. 9 with maximum difference of 0.8% for 6 MV and 0.9% for 15 MV. The largest difference between two linacs in electron applicator factors is 0.8% and that 6 cm × 6 cm and 25 cm × 25 cm present slightly higher differences compared to other field sizes.

The VMAT‐plan dose delivery error among the linacs resulted in excellent agreement between the seven linacs and the reference linac. All evaluation acheived 100% pass rate with 2 %, 2 mm critera, and ≥99.2% pass rate with 1%, 1 mm criteria.

## 4 DISCUSSIONS

The symmetry calibration resulted in values which are substantially lower than the vendor acceptance criteria (2%) (Fig. 2). Moreover the average symmetry decreased from 1.2% before calibration to 0.5% for photons, and from 1% to 0.5% for electrons. Therefore, it is recommended that the symmetry should be optimized beyond the vendor acceptance criteria thereby accomplishing higher degree of matching among the linacs, and values within 1% should be achieved. Furthermore, optimizing symmetry values is of a practical advantage clinically because it requires less frequent maintenance intervention. The photon symmetry results are an improvement compared to Gao et al.^24^ who presented symmetry values for seven Varian C‐series and four TrueBeam linacs below 1.1%. The electron symmetry results are also lower than Gao et al.^24^ who reported maximum symmetry value of 1.2%. These findings resulted in warning and action tolerance levels of symmetry values in the annual quality controls of 1% and 1.5%, respectively.

The vendor acceptance criteria for energy‐matching is 0.5% difference from the reference value specified for the depth dose at 10 cm (D10). In this work, diagonal profiles of seven linacs are compared with the reference linac and setting the same tolerance of 0.5%. The results are all within the set tolerance except for 15 MV which exceeds this limit in the region outside 15 cm from the central axis (Fig. 3). This is attributed to the effect of the flattening filter for which small inconsistencies among the linacs affect the beam. Consequently, priority was given to match linacs at the central axis which offers higher clinical advantages due to treatments often are conducted in fields <30 cm × 30 cm, which resulted in higher discrepencies near the penumbra. The vendor acceptance criteria for electron energy‐matching is specified as the difference between the reference and measured value of R90, R80 and R50. The criteria differs depending on the parameter: most strictly is 0.5 mm for R80 and 6 and 9 MeV. In this work, it is presented that 0.1 mm difference between the two linacs for all energies (Table 2) is achievable. This is lower than results reported from Glide‐Hurst et al.^11^ who presented maximum difference of 0.4 mm in R90 and R50 between two beam‐matched TrueBeam linacs. Tuning R90 and R80 between the linacs reduces systematic errors in dose delivery of clinical treatments. R50 is used to define *k_Q,Q0_* and *z_ref_* of the the electron beam according TRS‐398.^16^ Therefore, having an optimized R50 between the two linacs reduces systematic errors when applying the same beam data in the treatment planning system and simplify routine QA procedures using the same *k_Q,Q0_* and *z_ref_*.

The disparity in DLG (Fig. 5) before the calibration is due to installer‐to‐installer differences in setting up the linac. Therefore, it is essential to measure and adjust the DLG in the commisionging process and consequently beam‐matching. The DLG calibration reduced the maximum difference among the linacs from 39.9% (0.664 mm) to 2.3% (0.032 mm). Mihailidis et al.^25^ reported 1.3% difference in DLG between two beam‐matched TrueBeam linacs. However, the difference in number of linacs between the two studies explains the larger difference in this work. The relationship between the Gap value and DLG (Fig. 5) shows that differences in the Gap value in the order of 1/100th mm significantly affect the DLG. With a maximum difference of 0.032 mm resulting in the clinic setting a warning tolerance for the DLG to the nominal value plus/minus 0.07 mm.

The largest difference in field size among the linacs is 0.4 mm and the largest junction jaw offset is 0.2 mm (Table 4). Hernandez et al.^23^ reported field junction‐dosages between −4.5% and 5.2% which is higher than our results where the field junction dosages are between −2.5 and 1.2%. However, this is presumably due to difference in linac models of the studies. Essentially, both agree on EPID being a more suitable method of jaw calibration than light field method.

The largest difference in TPR_20,10_ measurements was 0.5 % (Table 5) confirming the energy‐matching of the linacs to same level as presented earlier. Comparison of PDDs among the linacs (Fig. 7) reveals higher discrepancies near the build‐up region which is why the comparison is performed from depth of 1 cm, and is a well‐established issue of scattered electron contamination.^26^ Regions deeper than the build‐up depth are within 0.3% agreement among the linacs against the reference. The verification water profile measurement results of this work are in agreement with Chang et al.^12^ who reported the mean standard deviation (SD) of PDDs and lateral dose profiles of 0.12% and 0.40%. respectively. Chang et al^12^ also reported a mean SD of 0.39% for electron PDDs. These OF verification results (Fig. 8) are in agreement with Beyer et al.^1^ who presented similar comparison results among three TrueBeam linacs for 10 cm × 10 cm, 20 cm × 20 cm, and 40 cm × 40 cm and 6, 15, and 10 MV FFF. The Wedge factor verification results (Fig. 9) are consistence with Glide‐Hurst et al.^11^ who reported the largest applicator factor difference for 6 cm × 6 cm and 12 MeV beams.

The verification of dose delivery error using a clinical VMAT‐plan measured on the Delta4 Phantom confirms the beam‐matching among the linacs to a high degree of precision. The output for each linac‐energy combination was normalized to an open field, allowing the measurement to isolate the effects of energy‐matching and field size‐ and DLG consistency. Considering that the majority of institutions implement 95%‐threshold in verifying their dynamic plan dose delivery against predicted TPS dose using gamma index evaluation with 3%, 3 mm criteria, our result can be interpreted as demonstrating extremly well‐tuned linacs. There is a lack of comparable measurements in the literature.

## 5 CONCLUSIONS

Energy matching and symmetry‐, DLG‐ and jaw calibration for TrueBeam linacs was performed with a high degree of precision, surpassing vendor acceptance criteria and international recommendations and was achievable within a reasonable time‐frame. The resulting beam matching reduced systematic errors in dose delivery and increased the confidence in using the same beam data in the TPS and swapping patients among linacs.

## CONFLICT OF INTEREST

No conflict of interest.

## AUTHORS' CONTRIBUTION STATEMENT

All authors planned and performed the measurements and the analysis. T.P. wrote the Matlab code and, together with M.G., created the figures. M.G. drafted the manuscript under the supervision of T.P. and input from L.S., M.W., and J.S. All authors approved the final version of the manuscript.

## Figures and Tables

**Fig. A1:**
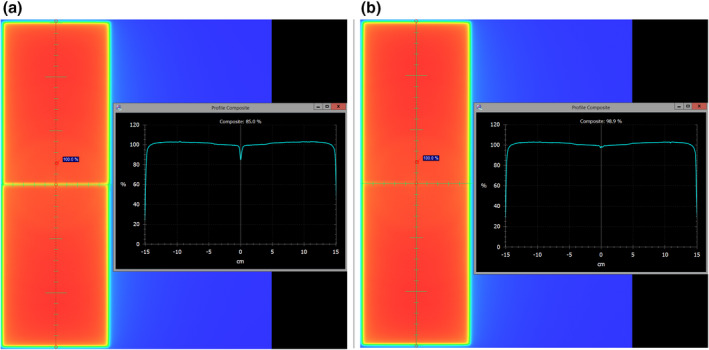
View of the Portal Dosimetry where two merged 6 MV EPID‐images are evaluated in the field junction resulting in (a) underdosage (before jaw calibration), (b) acceptable junction‐dosage (after jaw calibration).

**Fig. A2:**
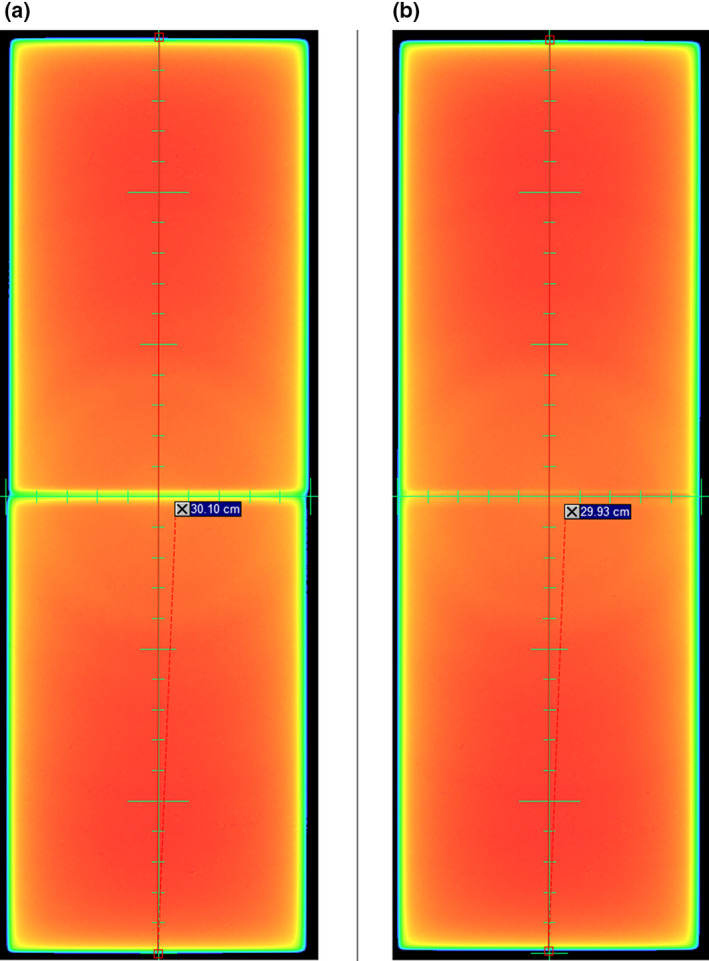
View of the Portal Dosimetry where two merged 6 MV EPID‐images are evaluated in terms of field size resulting in (a) too large field size (before jaw calibration), (b) acceptable field size (after jaw calibration). Note that the measured values in the figure understimate the field size by 0.07 cm yielding an actual field size of 30.17 and 30.01 cm in (a) and (b), respectively.

## Appendix A

### Calibration of jaws using EPID

The linac is equipped with two pairs of jaws: X1 and X2 in the crossline direction and Y1 and Y2 in the inline direction. The workstation provides position sensor readout (PRO‐value). Preparatory measurement for developing and implementing the EPID‐method revealed an underestimation of field size as determined by EPID of 0.7 mm on average, compared to water measurements. This underestimation was accounted for and is denoted CORR_EPID_. The following step‐by‐step method follows the calibration of the X‐jaws. The Y‐jaw calibration follows identical procedure.

#### Step 1: vertical position of EPID

Before each calibration, the EPID positioning in the vertical direction was visually verified using the SSD optical scale (98.8 cm to the surface equalling 100 cm to the detectors).

#### Step 2: evaluation of the jaws at two nominal positions

The two nominal positions for which the jaws are evaluated are: zero cm and 15 cm.

X1 is placed at zero position (at the junction) and X2 is placed at 15 cm. Two images are acquired with the EPID with 180° collimator rotation apart. The two images are merged in the Portal Dosimetry.

The X1 zero cm position is evaluated as follows:

- A profile is drawn over the junction area of the merged image to show any over‐ or underdosage which is recorded and denoted *CAX_X1*. This reveals the direction for which the jaw position should be corrected [Fig. A1(a)].
- Using the tool to align an image, one of the images is moved so that the over‐ or underdosage is corrected. The magnitude of movement is recorded and denoted *MOVE_X1*. Note that it should be recorded as an absolute value since the CAX_X1 value determines in which direction the jaw should be corrected.
- If *CAX_X1* is larger than 100 % → ‐ *MOVE_X1*/2 which is the correction for the nominal value (zero cm).

If *CAX_X1* is smaller than 100 % → *MOVE_X1*/2 which is the correction for the nominal value (zero cm).

The *MOVE_X1* value is divided by 2 because the correction is based on two images. The correction is denoted *X1_0_Corr_*.

The X2 15 cm position is evaluated as follows:

- The field size in the X direction of the merged image is measured at the 50 % isodose level corresponding to the full width half maximum which is commonly used to defined the field size [Fig. A2(a)]. It is recorded and denoted *FS_X2_measured_*.
- The correction of the nominal value (15 cm) is: (*FS_X2_measured_* + *CORR_EPID_*)/2 and is denoted *X2_15_Corr_*.

*CORR_EPID_* is added to correct for the underestimation of field size by EPID and the *FS_X2_measured_* value is divided by 2 because the correction is based on two images.

Similarly, X2 is placed at zero position and the X1 is placed at 15 cm. Two images are acquired with the EPID with 180° collimator rotation apart and evaluated using the same steps as described above. This will result in *X2_0_Corr_* and *X1_15_Corr_*.

#### Step 3. Calibration of the jaws

Each jaw is calibrated separately. The calibration mode of the jaws at the workstation is set at nominal positions of 1 and 19 cm.

- A linear fit is modeled between zero cm and 15 cm, and *X1_0_CORR_* and *X1_15_CORR_*. This is performed to predict the corrected position of the jaws at 1 and 19 cm denoted *X1_1cm_* and *X1_19cm_*, respectively.
- The PRO‐values are recorded at nominal position 1 and 19 cm, and denoted *PRO_X1_1cm_* and *PRO_X1_19cm_*, respectively.
- A linear fit is modelled between *X1_1cm_* and *X1_19cm_*, and *PRO_X1_1cm_* and *PRO_X1_19cm_*.

This is performed to predict the corrected PRO‐values for jaw positions at 1 and 19 cm denoted *PRO_X1_1cmCORR_* and *PRO_X1_19cmCORR_*.

- *PRO_X1_1cmCORR_* and *PRO_X1_19cmCORR_* are inserted in the workstation and the jaws should be re‐initialized.
- Step 2 should be repeated to verify successful calibration [Figs. A1(b) and A2(b)].
